# Omeprazole Inhibits Cell Proliferation and Induces G0/G1 Cell Cycle Arrest through Up-regulating miR-203a-3p Expression in Barrett’s Esophagus Cells

**DOI:** 10.3389/fphar.2017.00968

**Published:** 2018-01-09

**Authors:** Yichao Hou, Qiang Hu, Jiao Huang, Hua Xiong

**Affiliations:** Division of Gastroenterology and Hepatology, Key Laboratory of Gastroenterology and Hepatology, Ministry of Health, State Key Laboratory for Oncogenes and Related Genes, Renji Hospital, School of Medicine, Shanghai Jiao Tong University, Shanghai Institute of Digestive Disease, Shanghai, China

**Keywords:** omeprazole, miR-203a-3p, Gli1, Barrett’s esophagus cell, proliferation

## Abstract

Existing data suggest that proton pump inhibitors (PPIs), particularly omeprazole, have significant anti-tumor action in monotherapy and or combination chemotherapy. Hedgehog (Hh) signaling pathway represents a leading candidate as a molecular mediator of Barrett’s esophagus (BE). Studies have indicated reduced miRNAs in BE progression, however, little is known about the latent anti-neoplasm effects of miRNAs in BE cells. Here, we investigated whether omeprazole could inhibit BE progression by regulating Hh pathway and explored the promising Hh-targeted miRNAs in BE cells. We conducted qRT-PCR and immunoblotting assay to evaluate the effects of omeprazole on the expression of Hh signaling components and miR-203a-3p in CP-A and CP-B cells. The promising target genes of miR-203a-3p were predicted by bioinformatics methods, and verified by luciferase assays and qRT-PCR. The effects of omeprazole on BE cell proliferation and cell cycle distribution were determined. The overexpression or silencing of miR-203a-3p was performed to test its anti-proliferative effects. Finally, rescue experiments that miR-203a-3p inhibitor alleviated the effects of omeprazole on decreasing the levels of Gli1 mRNA, protein and luciferase were performed. Mechanistic studies showed that omeprazole could inhibit the expression of Gli1 and the nuclear localization of Gli1. Moreover, we determined that omeprazole could selectively up-regulated the expression of miR-203a-3p, and Gli1 was a bona fide target of miR-203a-3p. miR-203a-3p inhibitor alleviated the suppressing effects of omeprazole on Gli1 luciferase activity, mRNA and protein level. The functional assay suggested that omeprazole could dose-dependently inhibit BE cell growth and induce cell cycle arrest in G0/G1 phase. Additionally, overexpression and silencing of miR-203a-3p in BE cells disrupted cell cycle progress, resulting in suppressing and accelerating cell proliferation, respectively. Taken together, these data provide a novel mechanism of potentially anti-neoplastic effects for omeprazole through modulation of miR-203a-3p expression and thus suppressing Hh/Gli1 signaling in BE cells.

## Introduction

Barrett’s esophagus (BE) is the main risk factor known for developing esophageal adenocarcinoma (EAC), with an estimated annual incidence of 0.5% (Drewitz et al., 1997; Titi et al., 2012). Over the last decades the incidence of BE and EAC has risen dramatically in the Western word (van Soest et al., 2005). However, the risk is increased to 7.4% each year in BE with high-grade dysplasia (HGD), and half of BE patients with HGD develops into invasive cancer in 5 years (Wani et al., 2009). This increased risk has contributed to the management of BE patients with HGD, specifically, annual or biannual surveillance for HGD and localized endoscopic ablation, radiofrequency or esophagectomy, which has relatively high operative mortality and complication rates (Spechler, 2002). Therefore, effective and safe therapies prophylaxis against from BE to EAC are urgently required to be developed.

It is recognized that proton pump inhibitors (PPIs), particularly omeprazole, are the most widely used drugs in the treatment of BE (Falk et al., 2012; Watson et al., 2013). They act as potent inhibitors of the gastric acid pump, which increase the PH in the stomach and clearly relieve reflux symptoms (Klinkenberg-Knol et al., 2000). In addition to targeting the gastric acid pump, PPIs may inhibit vacuolar-type H^+^ ATPase (V-ATPase) activity to affect cancer cells homeostasis and counter the malignant behavior of cancer cells, including proliferation, migration, invasiveness and drug resistance (Martinez-Zaguilan et al., 1993; Luciani et al., 2004; Sennoune et al., 2004; Huang et al., 2012). Moreover, several lines of evidence suggest that inhibition of proton pumps activity could deprive cancer cells of the acidic microenvironment, which in turn leads to cancer cell death (De Milito et al., 2007, 2010; Canitano et al., 2016). Additionally, cumulative reports have confirmed that PPIs display anti-tumor effects mainly through acid-inhibitory approach. However, publications rarely explored the underlying non-acid inhibitory mechanisms of PPIs against tumor progression.

On the other hand, esophageal Hedgehog (Hh) signaling is active in the early embryonic stage when the esophagus is lined by columnar epithelium. However, Hh signaling is inactivated after the embryonic columnar lining differentiates into stratified squamous epithelium (Litingtung et al., 1998). Abnormal activation of Hh signaling in adult tissues could induce the expression of genes, which may determine an intestinal phenotype in esophageal squamous epithelial cells and therefore contribute to the development of Barrett’s metaplasia (Wang et al., 2014). So Hh signaling pathway, which plays an important role in normal gut development, represents a critical candidate as a molecular mediator of BE (Katoh and Katoh, 2009; Wang et al., 2010). In mammals, Hh signaling is initiated by the binding of Hh ligands, Sonic (Shh), India (Ihh), or Desert (Dhh), to the Hh receptor Patched (Ptch). In the presence of Hh ligand, Ptch releases constitutive repression of the signal transducer protein smoothened (Smo), which then activates the cytoplasmic protein complex containing glioma-associated zinc finger transcription factor (Gli1) transcription factors, ultimately leading to nuclear translocation of the Gli1 proteins and activation of pathway (Lees et al., 2005). In addition, studies have shown that the pathological process of BE is closely related to the altered expression of miRNAs in tissues and specific expression signatures or panels, which can also be used to classify progressive mechanism from BE to BE with HGD, EAC (van Baal et al., 2013; Drahos et al., 2016). miRNAs are small non-coding RNAs of about 22 nucleotides in length and are differentially expressed in various tissues (Calin and Croce, 2006). Expression profiles of miRNAs in many cancer tissues have been elaborated, and it has been shown that dysregulated miRNA expression is a hallmark for tumor evolution and progression by regulating the expression of cancer-related genes (Zhang et al., 2017). In particular, there is some evidence that miR-203a-3p is related to the development of BE (Smith et al., 2010). In addition, studies have shown that the expression of miR-203a-3p is lower in BE than in normal esophageal epithelia (Fassan et al., 2013; Mallick et al., 2016). Furthermore, it could be a promising biomarkers for diagnosis of BE and identifying novel drug targets and therapies (Mathe et al., 2009; Revilla-Nuin et al., 2013). To identify more novel targets of miR-203a-3p in BE, we predicted its target mRNAs using computational algorithms. Interestingly, miR-203a-3p was found to bind to 3′-UTR of Gli1 mRNA, a critical transcription factor of Hh pathway. Thus, miR-203a-3p may be involved in the regulation of Hh/Gli1 in BE progression.

Based on the above information, we hypothesized that omeprazole could prevent BE progression by regulating Hh pathway. In this paper, the effect of omeprazole on the expression of Hh-related molecules and inactivation of Hh/Gli1 signal transduction was studied. Our data presented that omeprazole up-regulated miR-203a-3p to restrict cell proliferation and induce cell cycle arrest. Moreover, we determined Gli1 as a target gene of miR-203a-3p, and miR-203a-3p knockdown partially reversed the inhibitory function of omeprazole through up-regulating Hh/Gli1 expression. Taken together, the results of this study demonstrate that regulation of miRNA expression may represent another layer of mechanisms responsible for the clinical use of omeprazole in BE patients, and lay the foundation for studies in the future.

## Materials and Methods

### Cell Culture

Human immortalized BE cell lines CP-A (ATCC CRL-4027) with non-dysplastic metaplasia and CP-B (ATCC CRL-4028) with high-grade dysplasia were maintained in epithelial cell medium 2 (ScienCell, Carlsbad, CA, United States) (Peng et al., 2014) supplemented with 5% fetal bovine serum (ScienCell), 100 U/ml penicillin and 100 μg/ml streptomycin sulfate (ScienCell) and were cultured at 37°C in a humidified atmosphere of 5% CO_2_, and 95% air.

### Cell Treatment and Transfection

For cell proliferation and cell cycle assays, BE cells were individually treated in complete medium with either vehicle control dimethyl sulfoxide (DMSO) or increasing concentrations of omeprazole as indicated (Sigma, Molndal, Sweden), which was freshly prepared in DMSO (protected from light) right before use. DharmaFECT 1 transfection reagent (Thermo Scientific Dharmacon Inc.) was used for transfection. CP-A and CP-B cells seeded in 60-mm Petri dishes (Thermo Scientific Nunc^TM^) were transfected with either miR-203a-3p mimics or miR-NC; miR-203a-3p inhibitor and matched NC at 100 nM, which were purchased from Genepharm Technologies (Shanghai, China). The sequence of the miR-203a-3p mimics was 5′-GUGAAAUGUUUAGGACCACUAG-3′, 5′-AGUGGUCCUAAACAUUUCACUU-3′; the sequence of negative control (NC) was 5′-UUCUCCGAACGUGUCACGUTT-3′, 5′-ACGUGACACGUUCGGAGAATT-3′. The sequence of miR-203a-3p inhibitor was 5′-CUAGUGGUCCUAAACAUUUCAC-3′; the sequence of NC was 5′-CAGUACUUUUGUGUAGUACAA-3′.

### Bioinformatics Methods

The potential target genes of miR-203a-3p were predicted by employing several online programs with databases of different bioinformatic algorithms, such as miRCode and RNAhybrid. We predicted potential target genes of miR-203a-3p mainly based on combination condition between 3′-UTR binding sites of target genes and the seed region (an exact match to positions 2–8 of the mature miRNA followed by an ‘A’) of miR-203a-3p. Furthermore, the minimum free energy values of miR-203a-3p-target genes hybridization via RNAhybrid software needs to be calculated to evaluate the binding potential between miRNA and corresponding target genes.

### Total RNA Extraction and Quantitative Real-Time PCR (qRT-PCR)

Total RNA was extracted from CP-A and CP-B cells using Trizol reagent (Takara, Japan). For mRNA analysis, the first-strand cDNA was synthesized using a PrimeScript RT Reagent Kit (Takara, Japan) in accordance with the protocol of manufacturer. qRT-PCR was used for the detection of target gene expression by employing the SYBR Premix Ex Taq II (Takara, Japan) on an ABI StepOnePlus Real-Time PCR System (Applied Biosystems, Grand Island, NY, United States). miRNA expression was determined with qRT-PCR, to this end, the first-strand cDNA was synthesized using Mir-X^TM^ miRNA First-Strand Synthesis Kit (Takara, Japan) and qRT-PCR was conducted with SYBR Premix Ex Taq II (Takara, Japan) on an ABI StepOnePlus Real-Time PCR System (Applied Biosystems, Grand Island, NY, United States). Ct values for mRNA and miRNA were normalized to GAPDH mRNA and U6, respectively, which were used as endogenous controls. The comparative Ct (ΔΔCt) method was employed for the calculation of the relative expression of these genes. Therefore, expression was expressed as fold difference relative to that of the untreated or negative control cells. The primers for miRNA-203a-3p (GeneCopoeia, Inc., HmiRQP0305) and U6 (GeneCopoeia, Inc., HmiRQP9001) were purchased from GeneCopoeia. The mRNA primers used in the qRT-PCR are shown in **Table 1**.

**Table 1:** List of the qRT-PCR primers used in this study.

Primer name	Sequence (5′-3′)
Shh-Forward	CAGTGGACATCACCACGTCT
Shh-Reverse	CCGAGTTCTCTGCTTTCACC
Ptch1-Forward	GGCAGCGGTAGTAGTGGTGT
Ptch1-Reverse	CGGGTATTGTCGTGTGTGTC
Ptch2-Forward	GTGTGGTGGGAGGCTATCTG
Ptch2-Reverse	GGGTAGTGGCAGCATTGAAG
Smo-Forward	CTATTCACTCCCGCACCAAC
Smo-Reverse	CAGTCAGCCCACAGGTTCTC
Gli1-Forward	GAAGTCATACTCACGCCTCGAA
Gli1-Reverse	CAGCCAGGGAGCTTACATACAT
Gli2-Forward	TGTAAGCAGGAGGCTGAGGT
Gli2-Reverse	TGGATGTGCTCGTTGTTGAT
Gli3-Forward	TCAAACCCGATGAAGACCTC
Gli3-Reverse	GACTTCAGGCTCCTGTTTGC
GAPDH-Forward	GCATTGCCCTCAACGACCAC
GAPDH-Reverse	CCACCACCCTGTTGCTGTAG


### Western Blotting

Cells were lysed using RIPA lysis buffer (Beyotime, China) containing a protease inhibitor cocktail (KangChen, Shanghai, China) on ice. The protein concentration was determined using a BCA protein assay kit (Beyotime Institute of Biotechnology, China). Proteins were separated on 10 or 12% SDS-PAGE and transferred onto PVDF membranes. The membranes were blocked with 5% skim milk for 2 h and subsequently incubated overnight at 4°C with anti-Smo (1:1000, Rabbit, Abnova), anti-Gli1 (1:1000, Rabbit, Cell Signaling Technology), anti-Gli2 (1:500, Rabbit, Cell Signaling Technology), anti-Sufu (1:1000, Rabbit, Cell Signaling Technology), anti-Ptch2 (1:1000, Rabbit, Cell Signaling Technology), anti-Cyclin D1 (1:1000, Rabbit, Cell Signaling Technology), anti-Cyclin A2 (1:1000, Mouse, Cell Signaling Technology), anti-CDK2 (1:1000, Rabbit, Cell Signaling Technology), anti-CDK4 (1:1000, Rabbit, Cell Signaling Technology), anti-CDK6 (1:1000, Mouse, Cell Signaling Technology), anti-P18 (1:1000, Mouse, Cell Signaling Technology), anti-P21 Waf1/Cip (1:1000, Rabbit, Cell Signaling Technology), anti-P27 Kip1 (1:1000, Rabbit, Cell Signaling Technology), anti-GAPDH (1:3000, Mouse, Santa Cruz) and anti-Lamin A/C (1:1000, Mouse, Abcam). After extensive washing with TBST, the membranes were then incubated with HRP-conjugated secondary antibodies (goat anti-mouse or goat anti-rabbit IgG) (1:3000, KangChen, Shanghai, China) for 1 h at room temperature. Finally, signal on the membranes were detected using the ECL detection system (SuperSignal West Femto Maximum Sensitivity Substrate, Thermo Fisher Scientific, Rockford, IL, United States).

### Fractionation of Nuclear and Cytoplasmic Protein

Cells were fractionated into cytoplasmic and nuclear fractions with the CelLytic NuCLEAR Extraction Kit according to manufacturer’s instruction (Sigma, St. Louis, MO, United States). Cells were harvested and washed twice with ice-cold PBS followed by resuspension and lysis of cell pellet in a lysis buffer (1 × Lysis Buffer, 1% 0.1 M DTT solution and 1% protease inhibitor cocktail) on ice for 15 min. The cytoplasmic fraction was obtained by spinning at 10000–11000 × g for 30 s. The supernatants (cytoplasmic protein) were harvested and frozen at -80°C for use. The crude nuclei pellets were then resuspended in Extraction buffer containing DTT and protease inhibitor cocktail by vortexing at high speed for 15–30 min, and centrifuged for 5 min at 20000–21000 × *g* to harvest the supernatant (nuclear protein), which was snap frozen for further use. The efficiency of cytoplasmic and nuclear extraction were verified by immunoblotting with Lamin A/C and GAPDH antibodies, respectively.

### Cell Proliferation Assays

Cell proliferation was evaluated with CCK-8 assays (Dojindo, Japan). CP-A and CP-B cells were seeded onto 96-well plates at 2000 cells per well. After attachment, omeprazole or equal amount of DMSO, miRNA mimics and miRNA inhibitor with their corresponding NC were added to the cells. CCK-8 solution was added to each well at the indicated times and incubated for an additional 2 h at 37°C. Cell viability was calculated as OD value at 450 nm absorption with a microplate reader according to the manufacturer’s instructions.

### Cell Cycle Analysis

CP-A and CP-A cells were plated onto six-well cluster plates and cultured for 48 h before harvest and fixation overnight at -20°C with ice-cold 75% ethanol. For flow cytometric analysis, cells were centrifuged, wash twice with PBS and incubated with propidium iodide (PI) (BD Biosciences) protecting from light for 15 min, and for each sample, cell cycle distribution was determined by analyzing 10000 events with FACS Calibur (Becton Dickinson, United States).

### Dual Luciferase Assay

CP-A and CP-B cells were plated onto 96-well plates and cultured overnight before cotransfection with 2 ng pRL-TK and 20 ng Gli1-pGL3, a luciferase reporter driven by Gli1 promoter (Gli1 promoter regions, -979 to 33 nt) or the pGL3-Basic vector with FuGene transfection reagent (Promega). After transfection, cells were treated with omeprazole or DMSO. After 48 h, cells were harvested and the luciferase activity was determined using the Dual-Luciferase Reporter Assay Kit (Promega).

To construct an expression vector containing the Gli1 3′-UTR fused to the 3′-end of a luciferase reporter, a 219-bp fragment containing the predicted miR-203a-3p target sites was synthesized and ligated into the pmir-Glo-control vector (Promega, United States). The 3′-UTR of Gli1 containing one putative miR-203a-3p-binding site was amplified and cloned into a pmir-Glo control vector with the restriction endonucleases NheI/SalI. In the mutated fragment, eight bases were introduced into the predicted miR-203a-3p target sites. Cells were plated onto 96-well plates 24 h before treatments. After 48 h, cells were harvested and the luciferase activity was determined as described above. All results were expressed as the relative firefly luciferase activity normalized to Renilla luciferase activity.

### Statistical Analysis

Statistical analyses were carried out with the SPSS 17.0 software package (SPSS Inc., Chicago, IL, United States) and GraphPad Prism 6 (San Diego, CA, United States). Each experiment was repeated at least three times. The data were presented as the mean ± standard deviation (SD). Student’s *t*-test and one-way analysis of variance (ANOVA) were used for statistical analysis of the difference between the mean values of two groups and multiple groups, respectively. Multiple comparison between the groups was performed using *post hoc* Student–Newman–Keuls test (S-N-K). *P*-value < 0.05 was considered to be statistically significant.

## Results

### Omeprazole Decreases Gli1 Expression and Might Suppress Nuclear Localization of Gli1 in BE Cells

**FIGURE 1:**
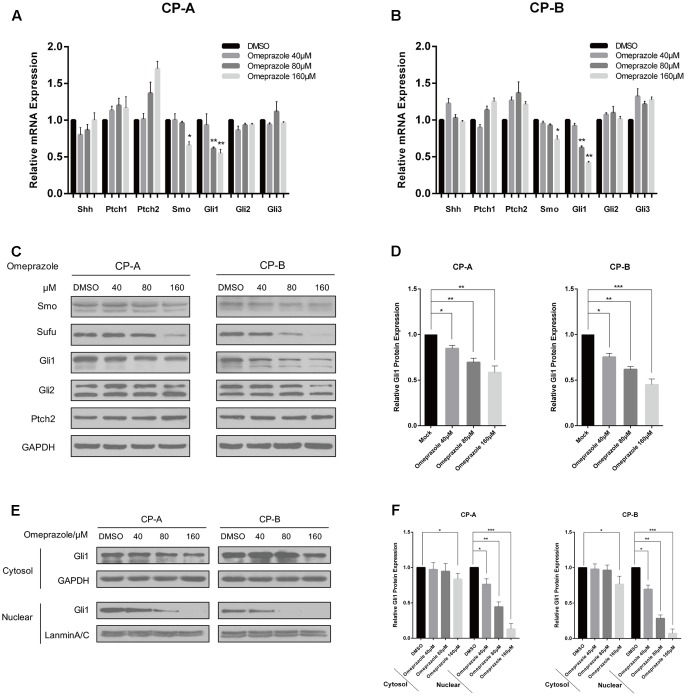
Omeprazole inhibits the expression of Gli1 and Gli1 nuclear localization in BE cells. **(A,B)** Expression of Shh, Smo, Ptch1, Ptch2, Gli1, Gli2, and Gli3 in CP-A and CP-B cells treated with increasing concentrations of omeprazole for 72 h; mRNA level of these genes was determined by qRT-PCR. **(C)** CP-A and CP-B cells were treated with DMSO or indicated concentrations of omeprazole for 72 h, and expressions of major molecules of Hh signaling were determined by immunoblot. A representative blot from three independent experiments is shown. **(D)** Quantitative results of Gli1 protein expression. **(E)** Nuclear and cytoplasmic proteins from CP-A and CP-B cells treated with DMSO or indicated concentrations of omeprazole for 72 h were isolated and the expression of Gli1 was determined by immunoblot. **(F)** Quantitative results of Gli1 protein expression. Each experiment was repeated three independent times, and data represent the mean of three experiments (mean ± SD). ^∗^*P* < 0.05, ^∗∗^*P* < 0.01 and ^∗∗∗^*P* < 0.001 vs. DMSO treated cells.

Previous studies demonstrated that Hh signaling pathway represents a leading candidate as a molecular mediator of BE. (Wang et al., 2010, 2014) Therefore, we studied the effect of omeprazole on the expression of Hh-related molecules, including Shh, Ptch1, Ptch2, Smo, Gli1, Gli2 and Gli3 in BE cells. After treatment with omeprazole for 72 h at 0, 40, 80, and 160 μM, the change in the Hh pathway molecules at mRNA level was determined by qRT-PCR. The results indicated that the mRNA levels of Smo and Gli1, indicators of the constitutive activation of Hh pathway, were reduced after treatment with omeprazole (**Figures 1A,B**). However, other components didn’t show any significant changes in mRNA levels in both BE cells. Subsequently, the protein levels of Hh-related genes were determined by western blot. Consistent with mRNA expression, the protein level of Gli1 was significantly decreased in omeprazole-treated BE cells. The other Hh-related genes, such as Smo, Sufu, Gli2, Path2, showed non-significantly change in dose-dependent manner in protein levels in both BE cells (**Figures 1C,D**).

Gli1 is the core transcription factor of Hh pathway. After activation, Gli1 translocates into the nucleus, and binds to promoter elements of responsive target genes to regulate their transcriptions. (Jiang and Hui, 2008) In this study, cytoplasmic and nuclear protein fractions were separated and the ability of omeprazole to decrease Gli1 level was also evaluated by Western blot. Our results indicated that omeprazole caused evident decreases in nuclear Gli1 levels in BE cells, which implied that omeprazole might modify Hh/Gl1 signaling further through suppressing nuclear localization (**Figures 1E,F**).

### Omeprazole-Induced Gli1 Down-regulation Is miR-203a-3p-Dependent

**FIGURE 2:**
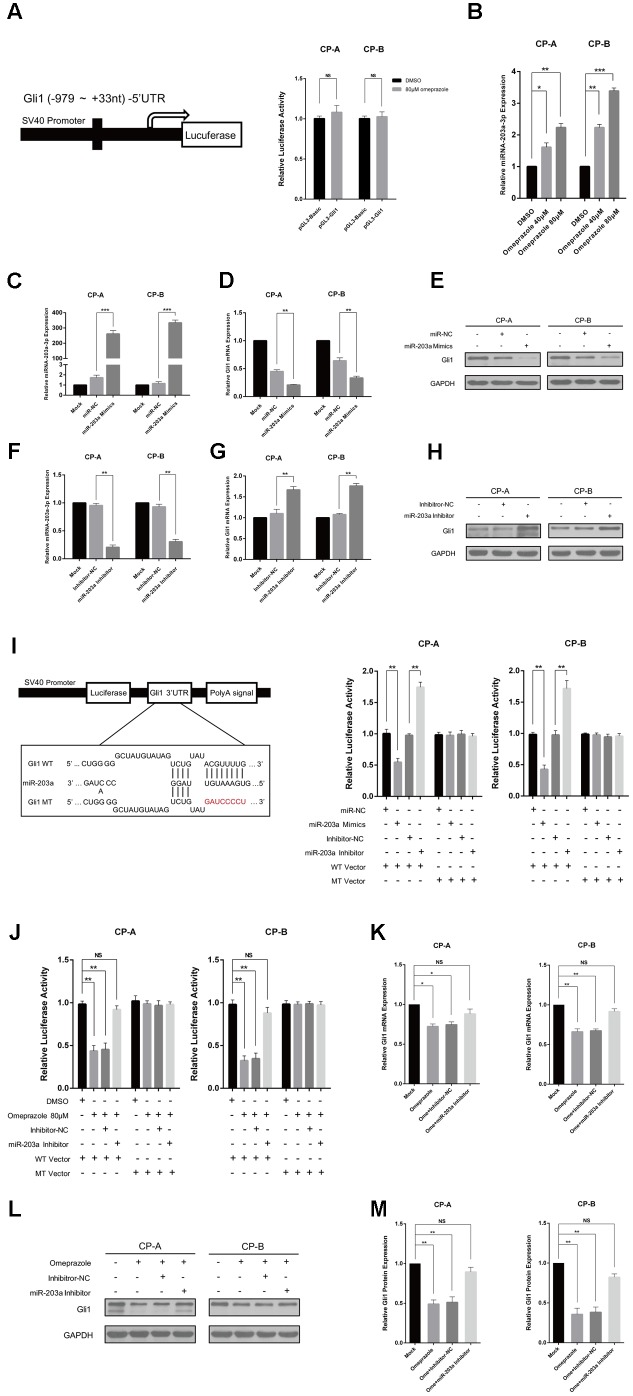
Omeprazole-induced Gli1 down-regulation is miR-203a-3p-dependent. **(A)** CP-A and CP-B cells were transiently transfected with pGL3-Gli1, pGL3-Basic vector and pRL-TK, followed by treatment with 80 μM omeprazole or DMSO. After 48 h, pGL3-Gli1 or pGL3-Basic vector reporter activities were measured and normalized to Renilla luciferase activity. **(B)** qRT-PCR was used to examine the expression of miR-203a-3p in CP-A and CP-B cells treated DMSO or indicated concentrations of omeprazole for 48 h. **(C–H)** Expression of miR-203a-3p and Gli1 of Hh signaling in BEs transfected with miR-203a-3p mimics or inhibitor was detected by qRT-PCR and immunoblot assays, respectively. **(I)** Sequences of miR-203a-3p and potential miR-203a-3p binding site at the 3′-UTR of Gli1. Direct biding of the miR-203a-3p to Gli1 3′-UTR. CP-A and CP-B cells were transfected using a firefly luciferase reporter containing either wild type (WT) or mutant type (MT) miR-203a-3p binding sites in the Gli1 3′-UTR with miR-203a-3p overexpression or knockdown. After 24h, cells were assessed using a luciferase assay kit. **(J)** CP-A and CP-B cells were transiently transfected with WT, MT Gli1 3′-UTR or pmir-Glo vector reporter, followed by treatment with DMSO, 80 μM omeprazole, omeprazole with inhibitor-NC or omeprazole with miR-203a-3p inhibitor. After 48 h, WT, MT Gli1 3′-UTR and pmir-Glo vector reporter activities were measured and normalized to Renilla luciferase activity. **(K–M)** CP-A and CP-B cells were transiently transfected with inhibitor-NC or miR-203a-3p inhibitor, followed by treatment with 80 μM omeprazole or DMSO. After 48 h, the expressions of Gli1 of were determined by qRT-PCR and immunoblot assays, respectively. Experiments were repeated three separate times, and each was performed in triplicate. Data represent the mean of three experiments (mean ± SD). NS (non-significance of difference), ^∗^*P* < 0.05, ^∗∗^*P* < 0.01, and ^∗∗∗^*P* < 0.001 vs. DMSO, miR-NC or inhibitor-NC treated cells.

To explore the mechanism by which omeprazole induced down-regulation of Gli1, we constructed the luciferase reporter plasmids, pGL3-Gli1, containing the promoter region of Gli1 (**Figure 2A**). Luciferase assay showed that 80 μM omeprazole had no effect on transcriptional activity of pGL3-Gli1 in BE cells, (**Figure 2A**) suggesting that omeprazole-induced inhibition of Gli1 was not dependent on the transcriptional suppression of Gli1 promoter.

miRNAs often regulate gene expression by binding to the RISC complex and directing sequence-specific cleavage of target mRNA or repressing the target mRNA translation (Krek et al., 2005). We wonder whether that dysregulated miRNAs may contribute to omeprazole-induced inhibition of Gli1 expression. Many articles have demonstrated several miRNAs play a vital role in the occurrence and development of BE, among which miR-203a-3p is a promising molecular biomarker for BE diagnosis (Bansal et al., 2011; Streppel et al., 2013; Ramzan et al., 2014). To investigate the responsiveness of miRNAs to omeprazole, we detected the miR-203a-3p expression after different concentrations of omeprazole treated with 0, 40, and 80 μM for 48 h. As shown in (**Figure 2B**), omeprazole significantly increased miR-203a-3p in both CP-A and CP-B cells, when compared with solvent only (negative controls).

Additionally, a bioinformatics analysis was carried out to identify the target genes of miR-203a-3p. We found that Gli1 3′UTR had binding sites of miR-203a-3p, suggesting that Gli1 was a potential target of miR-203a-3p (**Figure 2I**). Furthermore, the minimum free energy values of miR-203a-3p –Gli1 hybridization via RNAhybrid software was -15.3 kcal/mol. All these information imply that Gli1 might be a potential target of miR-203a-3p. To explore the relationship between miR-203a-3p and Gli1, we detected the roles of miR-203a-3p on Gli1expression. We detected Gli1 expression in CP-A and CP-B cells transfected with miR-203a-3p and its inhibitor, respectively, while the control cells were transfected with miR-NC or inhibitor-NC. As shown in **Figure 2C** the data showed that the expression of miR-203a-3p is increased in BE cells transfected with the miR-203a-3p mimics compared with the cells transfected with miR-NC. Moreover, the expression of miR-203a-3p was lower in BE cells transfected with the miR-203a-3p inhibitor than that in cells transfected with inhibitor-NC (**Figure 2F**). In line with the expression of miR-203a-3p, the level of Gli1 was down-regulated or up-regulated, as determined using qRT-PCR and western blot analysis, which indicated that Gli1 is a target gene of miR-203a-3p (**Figures 2D,E,G,H**). To demonstrate that Gli1 expression is regulated directly by miR-203a-3p, we sub-cloned the Gli1 3’-UTR containing the miR-203a-3p binding sequences [wild type (WT) or mutant (MT)] into a pmir-Glo vector. We first compared the luciferase activity between WT vector and MT vector. Results showed no statistical significance in the luciferase activity between WT vector and MT vector. We then transiently co-transfected the reporter construct with the miR-203a-3p mimics or inhibitor into CP-A and CP-B cells. The results indicated that miR-203a-3p could significantly inhibit luciferase activity in cells transfected with the WT Gli1 3′-UTR but had no effect on luciferase activity in cells transfected with the MT vector (**Figure 2I**).

To identify whether Omeprazole-induced Gli1 down-regulation is miR-203a-3p-dependent, luciferase assay was performed, and the results indicated that the luciferase activity was decreased significantly in the omeprazole or omeprazole with inhibitor-NC treated cells. However, there was no significant difference in luciferase activity between cells transfected with mock control and miR-203a-3p inhibitor in the presence of omeprazole. In addition, as expected, the luciferase activity was not affected by omeprazole, omeprazole with inhibitor-NC and omeprazole with miR-203a-3p inhibitor in cells transfected with the MT vector (**Figure 2J**). These results strongly indicated that the putative miRNA binding site of Gli1 was responsible for the omeprazole-miRNA-mRNA interaction. In order to further explore potential mechanism of the omeprazole induced down-regulation of Gli1, BE cells were treated with mock control, 80 μM omeprazole and 80 μM omeprazole with miR-203a-3p or inhibitor-NC. qRT-PCR analysis showed that miR-203a-3p inhibitor rescued the effects of omeprazole on decreasing the levels of Gli1 mRNA (**Figure 2K**). In the line of mRNA levels, western blot showed a similar tendency, in which miR-203a-3p inhibitor alleviated the suppressing effects of omeprazole on Gli1 protein level (**Figures 2L,M**).

### Omeprazole and miR-203a-3p Presents Similar Trends in Affecting BE Cells Proliferation and Cell Cycle Distribution

**FIGURE 3:**
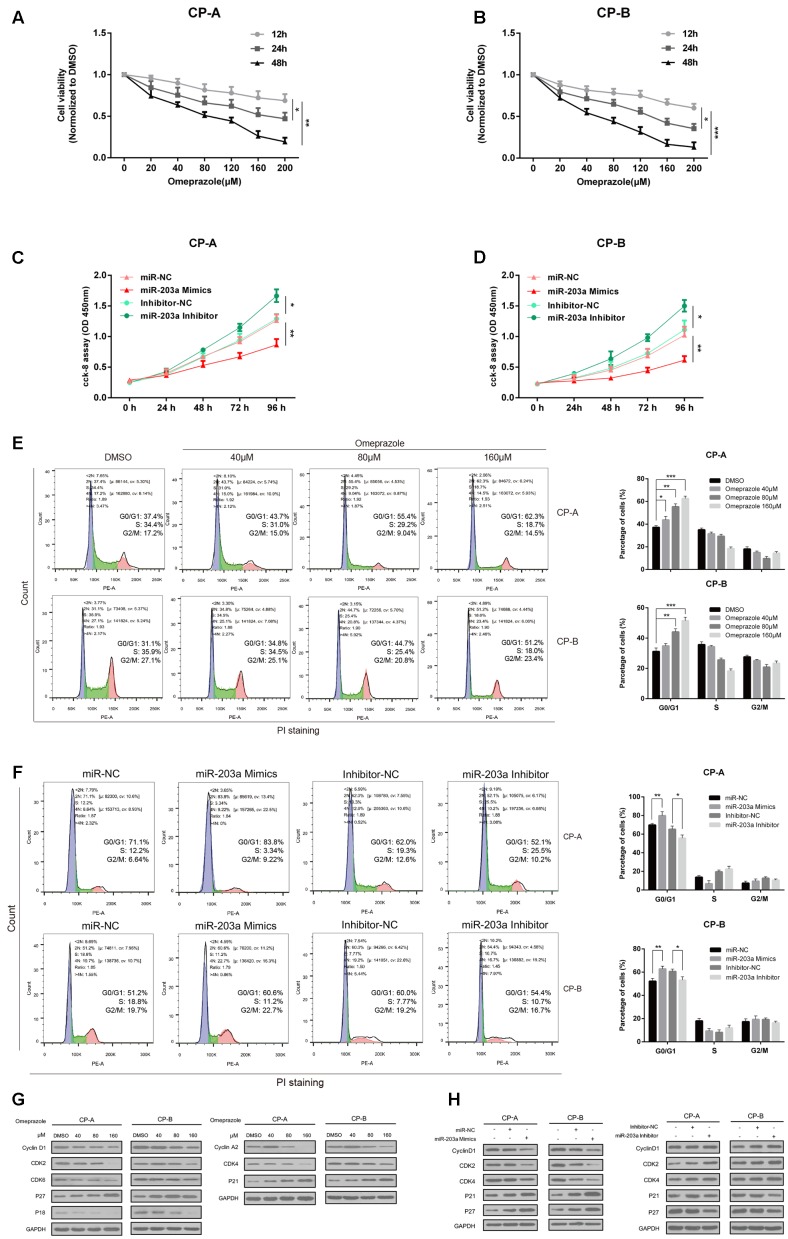
Omeprazole and miR-203a-3p presents similar trends in affecting BE cells proliferation and cell cycle distribution. **(A,B)** Analysis of cell proliferation using CCK-8 assay, CP-A and CP-B cells were incubated with omeprazole (0, 20, 40, 80, 120, 160, and 200 μM) for 12, 24, and 48 h. **(C,D)** CP-A, CP-B cells were transfected with miR-203a-3p mimics or miR-203a-3p inhibitor, and cell proliferation was determined by CCK-8 assay. **(E)** Cell cycle distributions of CP-A and CP-B cells treated with 0, 40 80, and 160 μM omeprazole are shown as flow cytometric histogram. **(F)** The cell cycle distributions of CP-A and CP-B cells were transfected with miR-203a-3p mimics or miR-203a-3p inhibitor for 48 h then detected by flow cytometry. **(G)** Expression of cell cycle-related proteins was examined by immunoblot assay, CP-A and CP-B cells were harvested after omeprazole treatment for 48 h. **(H)** The expression of cell cycle-related protein was examined by immunoblot assay, CP-A and CP-B cells were transfected with miR-203a-3p mimics or miR-203a-3p inhibitor for 48h. GAPDH was used as loading control. Experiments were repeated three separate times, and each was performed in triplicate. Data represent the average of three experiments (mean ± SD), ^∗^*P* < 0.05 and ^∗∗^*P* < 0.01 vs. DMSO, miR-NC or inhibitor-NC treated cells.

To evaluate the roles of omeprazole on BE cells proliferation, CCK-8 assay was performed in CP-A and CP-B cells. As shown in **Figures 3A,B**, omeprazole induced a decrease in the number of viable CP-A and CP-B cells in a dose-dependent manner after treatment for 12, 24, and 48 h. Notably, the concentrations of omeprazole in these experiments was more than 10-times higher than plasma concentration in clinical use, which suggested that V-ATPase was not mediated.

To investigate the underlying mechanisms responsible for the decrease in cell viability, the cell cycle distribution in response to omeprazole treatment was analyzed. We found that omeprazole treatment could result in a significant redistribution of cell cycle phases, manifested as a statistically significant increase in cell number in G0/G1 phase and a decrease in number in S phase when compared with control group (DMSO treatment) in CP-A and CP-B cells (**Figure 3E**). In CP-A cells, for example, the percentage of cells in the G0/G1 phase increased from 37.4% (vehicle control) to 43.7, 55.4, and 62.3% when cells were treated with 40, 80 and 160 μM omeprazole, respectively. Furthermore, the expression of cell cycle-related proteins, including cyclin D1, CDK2, CDK4, CDK6, cyclin A2, P18, P21Waf1/Cip1(P21) and P27KIP1(P27), was changed after omeprazole treatment for 48 h (**Figure 3G**). Among these proteins, omeprazole treatment dramatically inhibited the expression of cyclin D1, CDK2, CDK4 and increased the expression of P21Waf1/Cip1(P21) and P27KIP1(P27) in a dose-dependent manner, further indicating omeprazole induced G0/G1 phase arrest in CP-A and CP-B cells.

To study the potential function of omeprazole-regulated miRNA in BE cells, we overexpressed the miR-203a-3p by using miR-2013a-3p mimics and silenced miR-203a-3p using the miR-203a-3p inhibitor in BE cells and explored the effects on cell growth. CCK-8 assays showed that BE cells overexpressing miR-203a-3p had significantly lower proliferating ability than the control cells (**Figures 3C,D**). On the contrary, silencing of miR-203a-3p generated a significantly higher proliferating rate than that of control transfected cells (**Figures 3C,D**).

In addition, we continued to verify whether miR-203a-3p affected cell cycle progression. Cell cycle analysis showed that overexpression of miR-203a-3p led to a statistically significant increased cell number in the G0/G1 phase and a decreased cell number in the S phase compared to miR-NC group in CP-A and CP-B cells. Cells transfected with the miR-203a-3p inhibitor showed the opposite results (**Figure 3F**). We further determined the protein expression of some members of cell cycle signaling in miR-203a-3p overexpressing cells and found that Cyclin D1, CDK2 and CDK4 were decreased while P21Waf1/Cip1(P21) and P27KIP1(P27) were increased. In contrast, cells treated with the miR-203a-3p inhibitor revealed the opposite results (**Figure 3H**).

## Discussion

**FIGURE 4:**
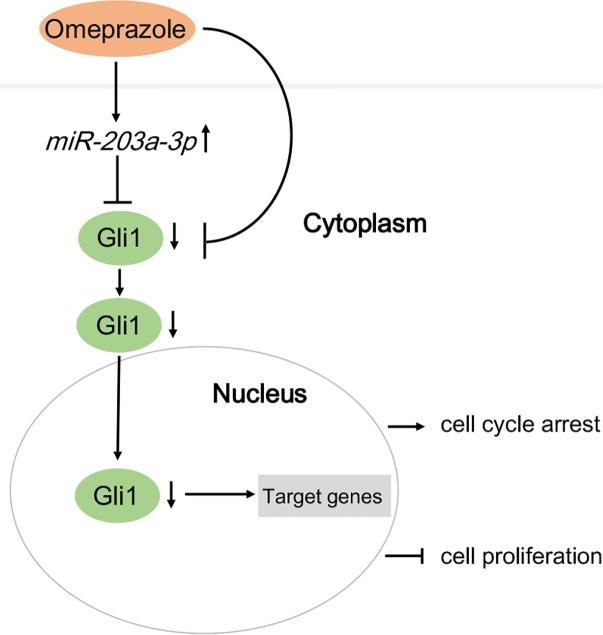
A model demonstrating how omeprazole displays anti-proliferation effects and induces cell cycle arrest via miR-203a-3p down-regulation of Hedgehog/Gli1 signaling.

The chemopreventive and anti-neoplastic effects of omeprazole are currently being evaluated for treatment of various tumors, including gastric cancer, human multiple myeloma and human B-cell tumors. (De Milito et al., 2007; Canitano et al., 2016; Kim et al., 2017) Although its mechanism of action is not fully understood, omeprazole is thought to generate anti-tumor effects locally through controlling cellular pH mediated by V-ATPase (Nishi and Forgac, 2002). In the present study, however, we investigated the mechanism underlying the anti-tumorigenic effect of omeprazole in BE cells from non-acid inhibitory view. Our findings showed that omeprazole inhibited Hh/Gli1 signaling and nuclear localization of Gli1 in CP-A and CP-B cells through up-regulating miR-203a-3p expression instead of transcriptional inactivation of Gli1 promoter. Subsequently, we verified that Gli1 was a target gene of miR-203a-3p. In addition, we observed that omeprazole inhibited the growth and induced the G0/G1 phase arrest in two BE cell lines. We also demonstrated that overexpression of miR-203a-3p in BE cells inhibited cell proliferation and induced cell cycle arrest in G0/G1 phase. Thus, omeprazole might function as a novel miR-203a-3p activator for both the treatment and prevention of BE to EAC through inhibiting Hh/Gli1 signaling (**Figure 4**).

It is generally believed that multiple factors are involved in the occurrence and development of BE, among which some miRNAs have a role in the formation of the neoplasia, and may become promising biomarkers as disease diagnosis and prognosis for BE. Several miRNAs that are highly expressed in BE are also expressed in higher levels in BE with HGD or EAC than in normal esophageal squamous mucosa. miR-203a-3p, being expressed at a lower level in BE than in normal esophageal epithelia, has been studied as a specific miRNA with promising biomarker potential for the diagnosis of BE and detection of BE, BE with HGD or EAC (Fassan et al., 2013; Revilla-Nuin et al., 2013; Slaby et al., 2015; Mallick et al., 2016). Most of these observations merely suggest that esophageal epithelial miRNAs may be only as biomarkers to identify prevalent HGD or EAC in BE, however, it has been rarely suggested that miRNAs may also be serve as therapeutic targets for prevention of EAC. In previous studies, miR-203a-3p has been found to be one of the miRNAs that are down-regulated in various types of solid tumors, such as colorectal cancer, gastric cancer, cervical cancer and osteosarcoma (Liu and Feng, 2015; Han et al., 2016; Yin et al., 2016; Gao et al., 2017). In addition, miR-203a-3p links to the tumor progression and functions as a tumor suppressor by inhibition of cell proliferation, migration and invasion. Further, in an elegant study conducted by Han et al., miR-203 is significantly up-regulated by sodium butyrate in colorectal cancer cells; this implies that miR-203a-3p has some putative function in cancer and potentially anti-tumor effects (Han et al., 2016). However, the tumor suppressive role of miR-203a-3p in BE is poorly understood. In the present study, we have reported for the first time that miR-203a-3p could be induced by omeprazole, and plays an important role in inhibition of cell growth and affecting cell cycle progression in BE cells, suggesting a tumor suppressor potential of miR-203a-3p in BE cells.

The classical Hh pathway is critical for development, adult tissue repair and homeostasis, and tumor initiation (Briscoe and Therond, 2013). Upon binding to a Hh ligand (Shh, Ihh, Dhh), Ptch releases its suppression of Smo that in turn, initiates the activation of Gli1 transcription factor; therefore, Gli1 can be used to monitor the activation of Hh pathway. Previous studies suggested that activation of Hh signaling in esophageal squamous epithelial cells can induce Gli1 and its downstream target genes that determine an intestinal phenotype and may contribute to the occurrence and progression of BE (Wang et al., 2014). In the past, numerous reports suggest that Gli1 is a biomarker of invasion, metastasis, or prognosis in various types of cancer, including lung squamous cell carcinoma, glioblastoma, breast cancer, colorectal cancer and pancreatic adenocarcinoma (Wang et al., 2016, 2017; Zhang et al., 2016; Kasiri et al., 2017; Liu et al., 2017). In addition, previous reports pointed out that Gli1 knockdown could suppress cancer cell growth, invasion, colony formation and induce cell cycle arrest, which further validates Gli1 as an oncogene (Vestergaard et al., 2008; Long et al., 2016; Oladapo et al., 2017; Sun et al., 2017). In this study, we detected the Hh-related genes expression in BE cell lines. Among these genes, it was found that the expression of Gli1 was significantly down-regulated by omeprazole at 80 and 160 μM at mRNA and protein levels. Additionally, omeprazole could inhibit the nuclear localization of Gli1, which may represent another layer of mechanisms responsible for the regulatory effect of omeprazole on Gli1 and its target genes. On account that Gli1 acts as terminal foci for numerous other oncogenic pathways, whence omeprazole may display anti-tumor effects via its inhibitory effects on Hh/Gli1 signaling.

Although there is ample evidence for the up-regulation of Gli1 expression in human BE, the underlying mechanism is poorly understood. In present study, we focused on BE cells to determine whether miRNAs can epigenetically influence Gli1 expression. Intriguingly, the miR-203a-3p level was up-regulated by omeprazole. The results imply that miR-203a-3p functionally contributes to the expression of Gli1 in BE cells. Although several miRNAs, especially miR-203a-3p, have been implicated in colorectal cancer, gastric cancer, cervical cancer and osteosarcoma, the targets of miR-203a-3p have not been identified in BE. Therefore, in this study, we performed *in silico* prediction of miRNA targets and found that miR-203a-3p can potentially bind to 3′-UTR of Gli1. Indeed, ectopic expression of miR-203a-3p in BE cells demonstrated that there was a reverse relationship between the expression level of miR-203a-3p and that of Gli1. These results provide a strong rationale for our findings that miR-203a-3p inhibits BE cells growth and affects cell cycle progression by suppression of Gli1, which further testifies that miR-203a-3p/Gli1 mediate the anti-tumor effects of omeprazole in BE cells.

Taken together, these results are the first report that omeprazole can up-regulate miR-203a-3p to inhibit BE cell proliferation and induce G0/G1 phase arrest. We also identify Gli1 as a pathogenic gene and a bona fide target of miR-203a-3p in BE cell lines. Furthermore, it reveals that omeprazole displays anti-proliferation and induces cell cycle arrest mainly through selective up-regulation of miR-203a-3p expression and thus inhibiting Gli1 expression. Therefore, omeprazole may serve as a novel Hh/Gli1 inhibitor, and should be useful for further development of drugs targeting miR-203a-3p/Gli1 by employing omeprazole as the lead compound for treatment and chemoprevention of BE. In conclusion, our findings shed light on the potential mechanism underlying the process that omeprazole displays anti-tumor effects on BE cells.

## Author Contributions

HX participated in the research design. YH, QH, and JH conducted the experiments. YH performed the data analysis and contributed to the writing of the manuscript. All authors have read and approved the final manuscript.

## Conflict of Interest Statement

The authors declare that the research was conducted in the absence of any commercial or financial relationships that could be construed as a potential conflict of interest.
